# Supplementary material for: Bandwidth-control orbital-selective delocalization of 4f electrons in epitaxial Ce films
Source: Nat Commun. 2021 May 4;12:2520. doi: 10.1038/s41467-021-22710-2 (PMC8096960; doi:10.1038/s41467-021-22710-2)
Supplement: Supplementary file 1 — Supplementary Information [file 41467_2021_22710_MOESM1_ESM.pdf]

Supplementary information for:

Bandwidth-control orbital-selective delocalization of 4f electrons in epitaxial Ce films

Yi Wu[1], Yuan Fang[1], Peng Li[1], Zhiguang Xiao[1], Hao Zheng[1], Huiqiu Yuan[1,2,3], Chao Cao[4], Yi-feng Yang[*5,6,7] and Yang Liu[†1,2,3]

[1] *Center for Correlated Matter and Department of Physics, Zhejiang University, Hangzhou 310058,P.R. China*

[2] *Zhejiang Province Key Laboratory of Quantum Technology and Device, Zhejiang University, Hangzhou, China*

[3] *Collaborative Innovation Center of Advanced Microstructures, Nanjing University, Nanjing 210093 ,P.R. China*

[4] *Department of Physics,Hangzhou Normal University,Hangzhou,P.R. China*

[5] *Beijing National Laboratory for Condensed Matter Physics, Institute of Physics, Chinese Academy of Sciences, Beijing 100190, China*

[6] *School of Physical Sciences, University of Chinese Academy of Sciences, Beijing 100190, China*

[7] *Songshan Lake Materials Laboratory, Dongguan, Guangdong 523808, China*

[*] Corresponding author: yifeng@iphy.ac.cn

[†] Corresponding author: yangliuphys@zju.edu.cn

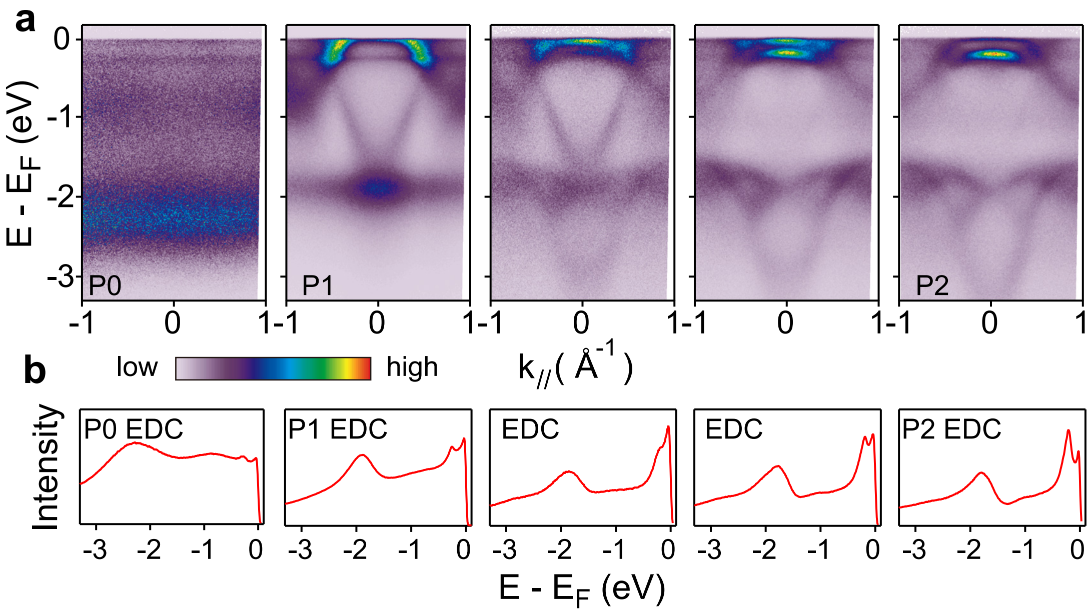

Supplementary Figure 1: **ARPES spectra of a thick Ce film taken at 20 K after sequential post-growth annealings from ∼370 K (P1) to ∼540 K (P2).** **a** ARPES spectra at various stages of annealing, showing the transition from P0, to P1 and finally to P2. **b** Corresponding EDCs at the $\bar{\Gamma}$ point. Note that the V-shaped conduction band at ∼-1.8 eV is still present in P2, although it is barely observable in the current color contrast.

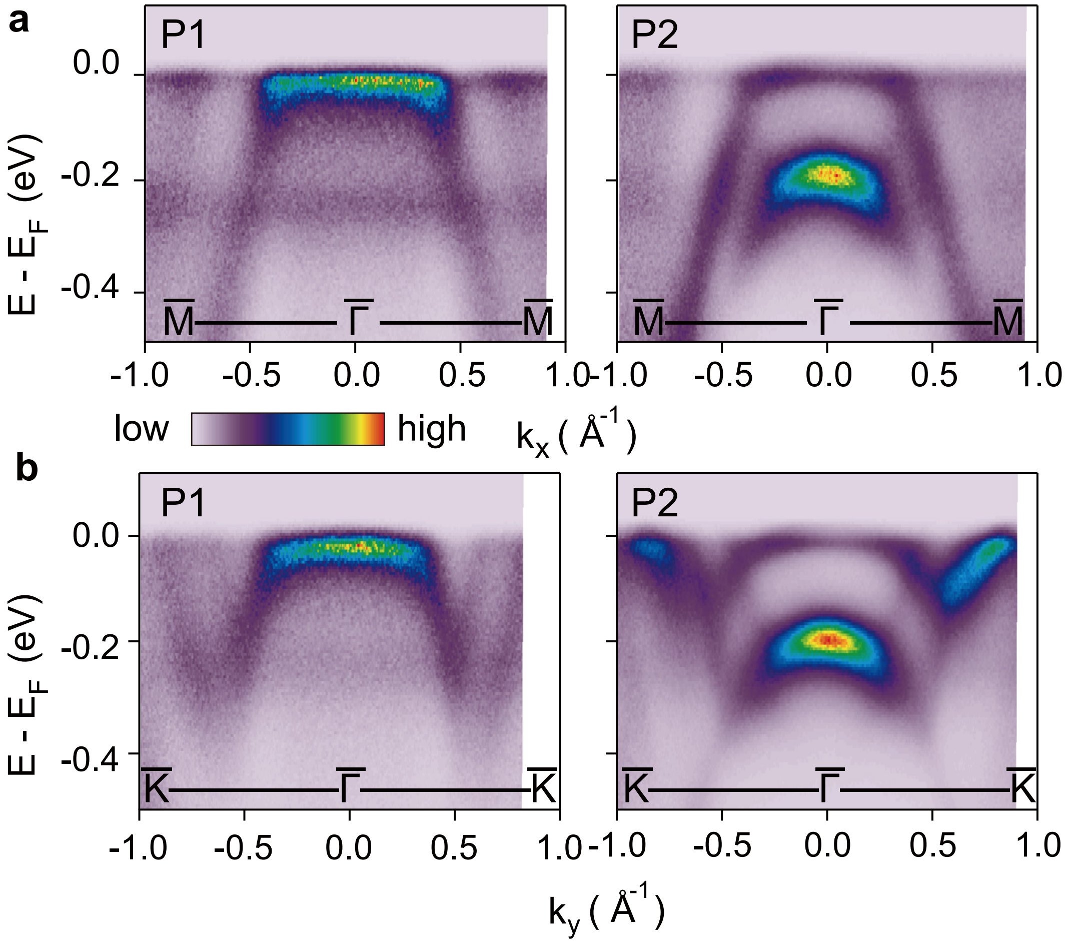

Supplementary Figure 2: ARPES spectra along different direction. **a** ARPES spectra along $\bar{\Gamma}\bar{M}$ for P1 (left) and P2 (right). **b** ARPES spectra along $\bar{\Gamma}\bar{K}$ for P1 (left) and P2 (right).

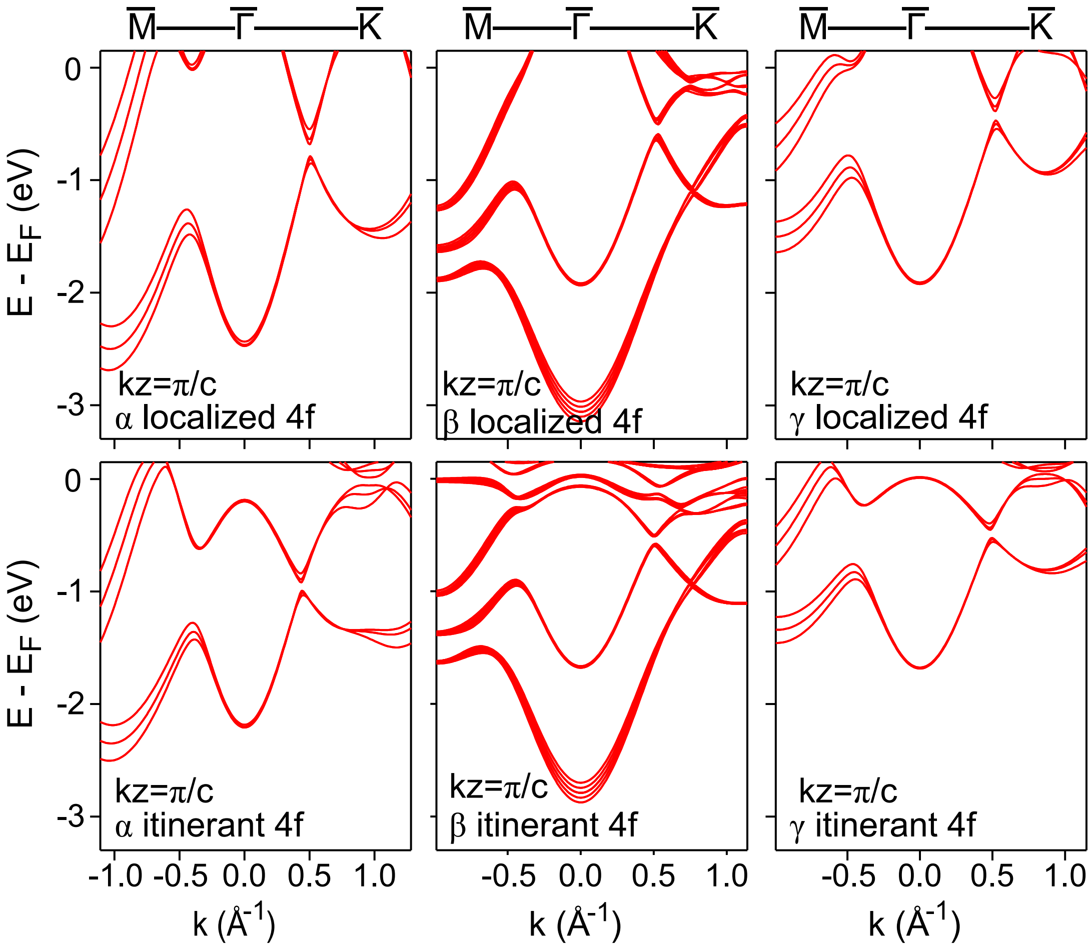

Supplementary Figure 3: **DFT calculation for bulk Ce.** DFT band structures of the α, β and γ phases of Ce assuming localized $4f$ electrons (top rows) and itinerant $4f$ electrons (bottom rows). From left to right are the calculation results for the α phase (left), the β phase (middle) and the γ phase (right). The in-plane momentum direction is along the $\bar{M} - \bar{\Gamma} - \bar{K}$, with $k_z = \pi/c$. A small k_z broadening (one tenth of the Brillouin zone) is included in the calculation to simulate the experimental results. The ARPES spectra of the P1 phase could be understood from the simple hybridized band picture within PAM: the conduction bands, well described by the localized $4f$ calculation for the γ phase (top right panel), hybridize with the Kondo resonance peaks near E_F, producing the characteristic hybridized bands observed experimentally (dashed green curves in Fig. 2c. For the P2 phase, the quasiparticle bands are well described by itinerant $4f$ calculations of the β phase (or the AB-stacking hcp phase, see Supplementary Figure 8), using the reduced lattice constant determined by ex-situ XRD in Fig. 5c, as discussed in the manuscript. We mention here that the $\gamma - \alpha$ transition is normally considered to be an isostructural transition with $\sim 5\%$ lattice constant reduction, i.e., the Ce-Ce distance changing from ∼3.64 Å to ∼3.41 Å. On the other hand, the Ce-Ce distances for bulk γ and β phases are identical ($\sim$ 3.64 Å) – the main difference is their layer stacking sequence, i.e., ABCABC stacking for γ and ABAC stacking for β.

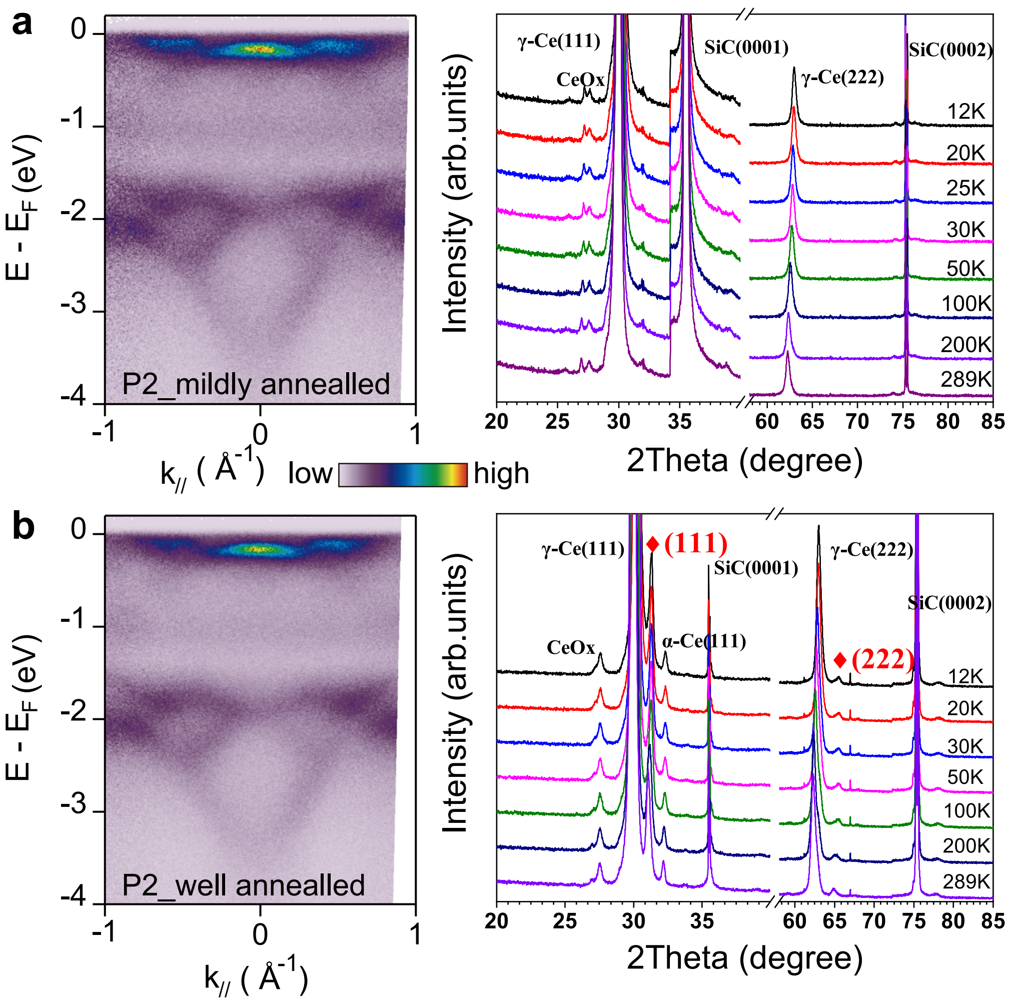

Supplementary Figure 4: **Ex-situ temperature-dependent XRD results for mildly annealed (a) and well annealed (b) P2 samples (thickness ∼200 nm).** Left panels are ARPES spectra taken at room temperature after growth, showing similar P2-type electronic structure; right panels are the corresponding ex-situ XRD results, with each peak labelled individually. While both types of samples show dominant diffraction peaks corresponding to the bulk γ phase, the well annealed samples show additional diffraction peaks at $\sim 31^\circ$ and $\sim 65^\circ$, corresponding to a metastable surface structure with a reduced interlayer spacing ($\sim$ 3.5% reduction compared to bulk γ), as discussed in the manuscript. These peaks are not observed in the mildly annealed samples, likely due to much smaller thickness. These results indicate that the metastable surface phase forms initially near the surface (detectable by surface-sensitive ARPES), grows with progressive annealing and eventually acquires sufficient thickness/volume to be detectable by bulk-sensitive XRD. Note that the tiny peak at $\sim 33^\circ$ is close to the (111) peak of the α phase, although its higher-order peaks are almost absent, implying very small volume (if indeed present).

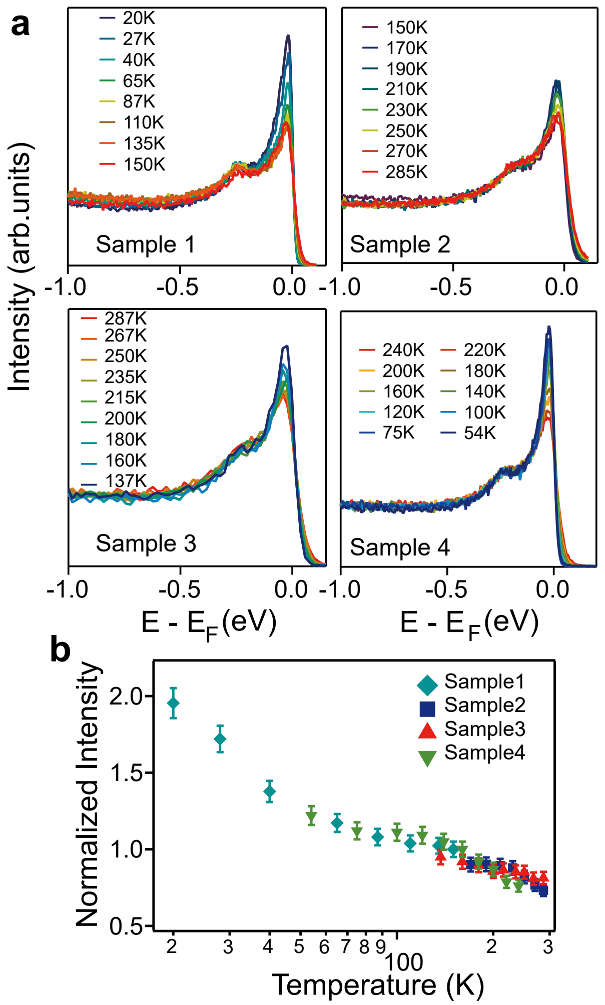

Supplementary Figure 5: **Temperature evolution of the Kondo resonance peaks for P1.** **a** Temperature-dependent EDCs at $\bar{\Gamma}$ from a few representative samples, covering different temperature ranges. **b** The extracted peak intensity as a function of temperature, normalized to the value at 150 K. The extracted intensity was obtained after subtracting the high energy background. We mention that due to the low counts from He-II photons and short in-vacuum life time of chemically reactive Ce (particularly for P1, which is < 4 hours), it is difficult to reliably obtain the temperature dependence of the Kondo resonance peaks from one single sample. Therefore, we used different samples that were grown under identical conditions, to cover different temperature regions. The intensity is normalized to the value at 150 K to get rid of sample variations. The peak intensity roughly follows the -log(T) behavior, as expected for a Kondo peak. The error bar is from the statistical noise of the experimental data.

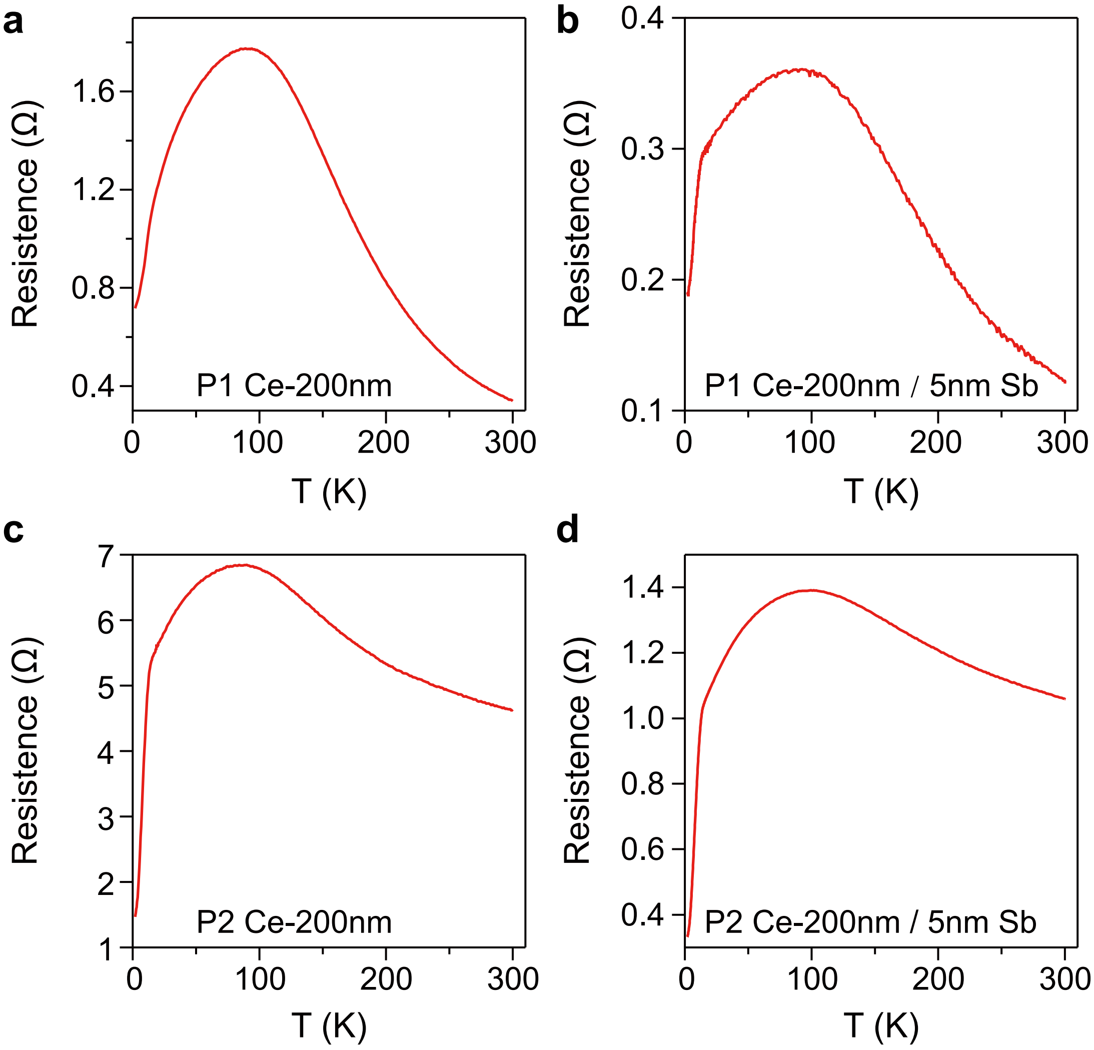

Supplementary Figure 6: **Ex-situ transport results of the P1 (a,b) and P2 (c,d) phases.** Measurements from uncapped samples (**a,c**) and those capped by ∼5 nm Sb amorphous films (**b,d**) are similar. The broad hump at ∼90 K, present both in P1 and P2, is likely due to the transport coherence, as seen in many Kondo lattice systems (note that a majority part of the film is γ-like for both cases, see Supplementary Figure 5). Despite showing a similar hump structure as P1, the resistivity of P2 exhibits stronger decrease with lowering temperature, likely due to more delocalized $4f$ electrons from the metastable surface phase in P2. The rapid decrease of the resistivity below ∼12 K could be attributed to a partial transition to the α phase as seen by low-temperature XRD (Fig. 5a), or the coexisting β phase (the β phase exhibits an antiferromagnetic transition near this temperature and it is a possible candidate for the metastable surface structure, as discussed in the manuscript). The transition at ∼12 K is irrelevant to our ARPES results, which are performed at ∼20 K and above.

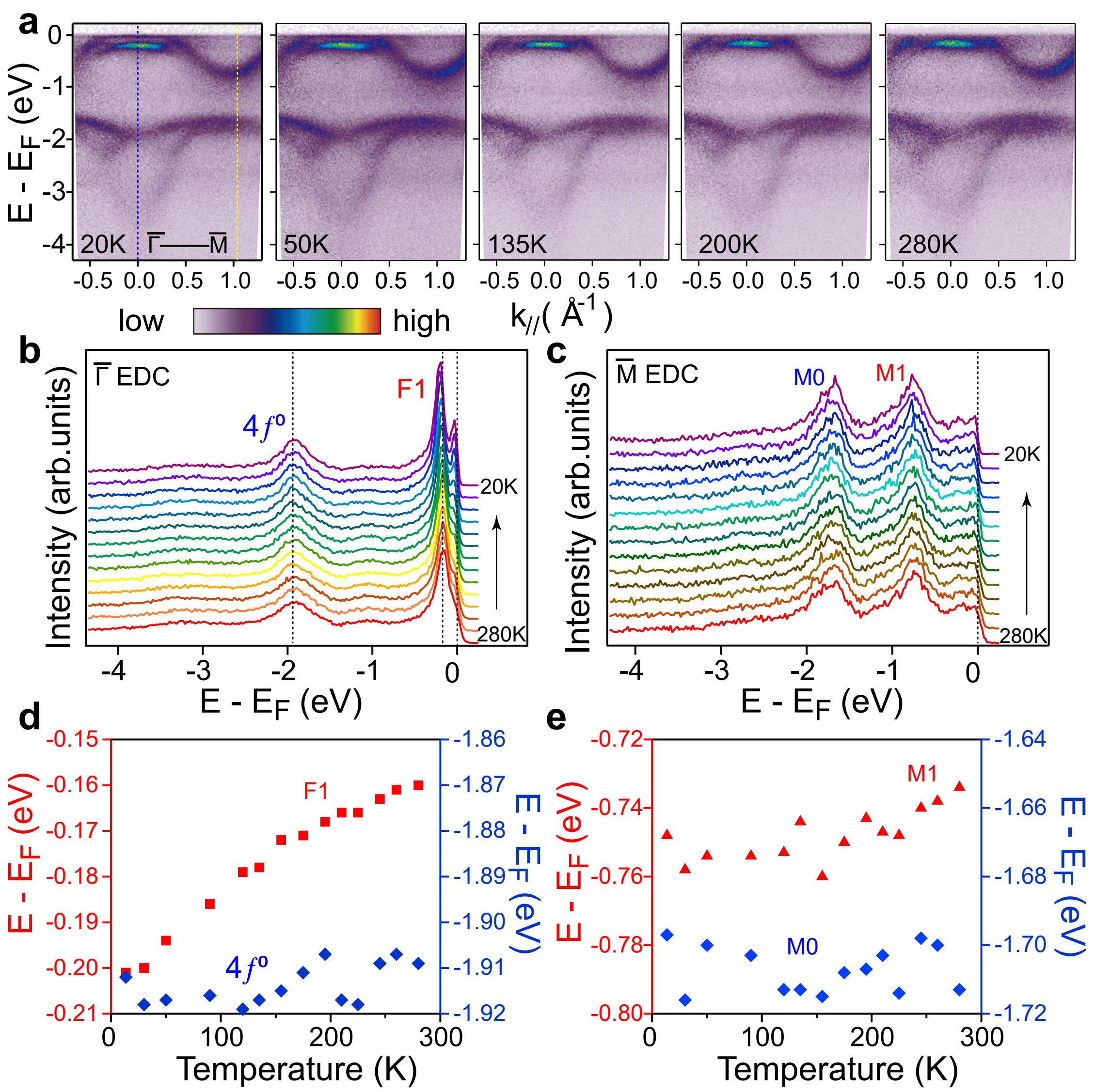

Supplementary Figure 7: **Analysis of the temperature-dependent electronic structure of the P2 phase. a** Temperature evolution of the energy-momentum dispersion along $\bar{\Gamma}\bar{M}$. Blue and yellow vertical dashed lines mark the $\bar{\Gamma}$ and $\bar{M}$ points. **b,c** Temperature-dependent EDCs at the $\bar{\Gamma}$ (b) and $\bar{M}$ (c) points. **d,e** Extracted peak positions as a function of temperature from EDCs in (**b,c**).

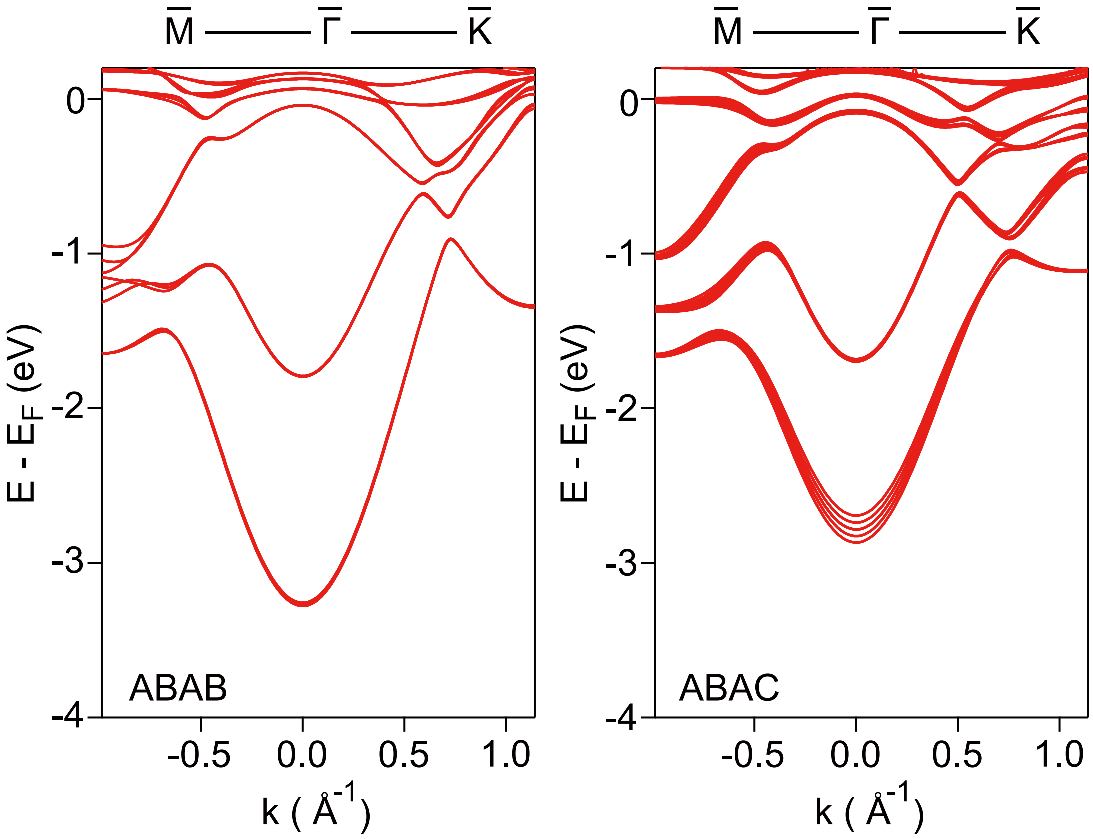

Supplementary Figure 8: **Comparison of itinerant $4f$ calculation for AB-stacking and ABAC-stacking hcp phases.** The results show that the overall electronic structures are not very sensitive to the layer stacking sequence and both can explain the experimental P2 phase.
